# TNFα Impairs Rhabdoviral Clearance by Inhibiting the Host Autophagic Antiviral Response

**DOI:** 10.1371/journal.ppat.1005699

**Published:** 2016-06-28

**Authors:** Raquel Espín-Palazón, Alicia Martínez-López, Francisco J. Roca, Azucena López-Muñoz, Sylwia D. Tyrkalska, Sergio Candel, Diana García-Moreno, Alberto Falco, José Meseguer, Amparo Estepa, Victoriano Mulero

**Affiliations:** 1 Departamento de Biología Celular e Histología. Facultad de Biología. Universidad de Murcia, IMIB-Arrixaca, Murcia, Spain; 2 Instituto de Biología Celular y Molecular, Universidad Miguel Hernández, Elche, Spain; University of Pennsylvania School of Medicine, UNITED STATES

## Abstract

TNFα is a pleiotropic pro-inflammatory cytokine with a key role in the activation of the immune system to fight viral infections. Despite its antiviral role, a few viruses might utilize the host produced TNFα to their benefit. Some recent reports have shown that anti-TNFα therapies could be utilized to treat certain viral infections. However, the underlying mechanisms by which TNFα can favor virus replication have not been identified. Here, a rhabdoviral infection model in zebrafish allowed us to identify the mechanism of action by which Tnfa has a deleterious role for the host to combat certain viral infections. Our results demonstrate that Tnfa signals through its receptor Tnfr2 to enhance viral replication. Mechanistically, Tnfa does not affect viral adhesion and delivery from endosomes to the cytosol. In addition, the host interferon response was also unaffected by Tnfa levels. However, Tnfa blocks the host autophagic response, which is required for viral clearance. This mechanism of action provides new therapeutic targets for the treatment of SVCV-infected fish, and advances our understanding of the previously enigmatic deleterious role of TNFα in certain viral infections.

## Author Summary

Tumor necrosis factor alpha (TNFα) is one of the main pro-inflammatory cytokines produced in response to a broad type of infections [1]. Although TNFα has a crucial role in protecting the host organism from pathogens, its deregulation can promote susceptibility to pathogens by impairing pathogen clearance and, ultimately, promoting maintenance of infection and death. In addition, some viruses might utilize the host produced TNFα to their benefit. Thus, anti-TNFα therapies could be utilized to treat certain viral infections. However, the underlying mechanisms by which TNFα can favor certain virus replication have not been identified. Here, we have used a viral infection model in zebrafish to identify the mechanism of action by which TNFα has a deleterious role for the host to combat certain viral infections. Our results demonstrate that Tnfa does not affect viral ability to infect host cells or to antagonize the main host antiviral pathway, namely the interferon pathway. However, Tnfa impairs viral clearance by blocking the host autophagy response, which is usually used by host cells to degrade unnecessary or dysfunctional cellular components, and that we found to be critical to eliminate intracellular viral particles. This mechanism of action provides new therapeutic targets for the treatment of SVCV-infected fish in aquaculture and probably to other viral infection affecting cattle industry and human.

## Introduction

Tumor necrosis factor alpha (TNFα) is one of the main pro-inflammatory cytokines produced in response to a broad type of bacterial, viral and fungal infections [1]. TNFα has a crucial role in activating and orchestrating the immune response in order to protect the host organism from pathogens. TNFα deregulation can promote susceptibility to pathogens by impairing pathogen clearance and, ultimately, promoting maintenance of infection and death. When specifically talking about viral pathogenesis, TNFα has been shown to inhibit the replication of certain viruses such as hepatitis B virus (HBV) and the varicella zoster virus (VZV) [2]. In addition, anti-TNF therapies to treat autoimmune diseases exacerbate the infection produced by virus such as herpes simplex virus (HSV), Epstein-Barr virus (EBV), cytomegalovirus (CMV) and human papillomavirus (HPV) [3]. It is not surprising that due to the key role of TNFα in the host protection to viral infections, some viruses have developed different ways to interfere with the TNFα pathway [4]. In contrast, it seems that a few viruses might utilize the host produced TNFα to their benefit. Interestingly, human immunodeficiency virus 1 (HIV-1) infection induces TNFα expression. These increased TNFα levels in serum correlates to increased viral replication [5]. In accordance to that, TNFα inhibitors are able to impair HIV-1 replication [6], and anti-TNFα treatments have been proposed to combat HIV-1 infection in combination with other therapies [7] [5]. Similarly, neutralization of TNFα decreases virus production in CMV-infected macrophages [8]. The ability of TNFα to favor virus replication has also been demonstrated for non-mammalian viruses, such as the spring viremia of carp virus [9], a fish rhabdovirus infecting cyprinids [10,11]. Moreover, intraperitoneally SVCV-infected adult fish, in which recombinant TNFα was administrated simultaneously, has shown a higher mortality rate than fish injected with the virus alone. The mechanism explaining how TNFα facilitates viral infection and its deleterious effects in the host has not yet been proposed.

Since zebrafish is a cyprinid susceptible to SVCV infection, and TNFα can exacerbate SVCV infection, we chose this amenable infection model to investigate how a virus might utilize host produced TNFα to their benefit. To that end, we analyzed the role of zebrafish TNFα (Tnfa) in i) the key steps of SVCV pathogenesis: virus adhesion, fusion, and replication; and ii) in the antiviral host response, such as interferon production and autophagy. The results showed that Tnfa signaling through its receptor Tnfr2 inhibits autophagy, leading to impaired viral clearance in SVCV-infected cells. This mechanism of action provides new therapeutic targets for the treatment of SVCV-infected fish, and advances our understanding of the previously enigmatic deleterious role of TNFα in certain viral infections.

## Results

### Tnfa increases susceptibility of zebrafish to SVCV infection

**Fig 1:**
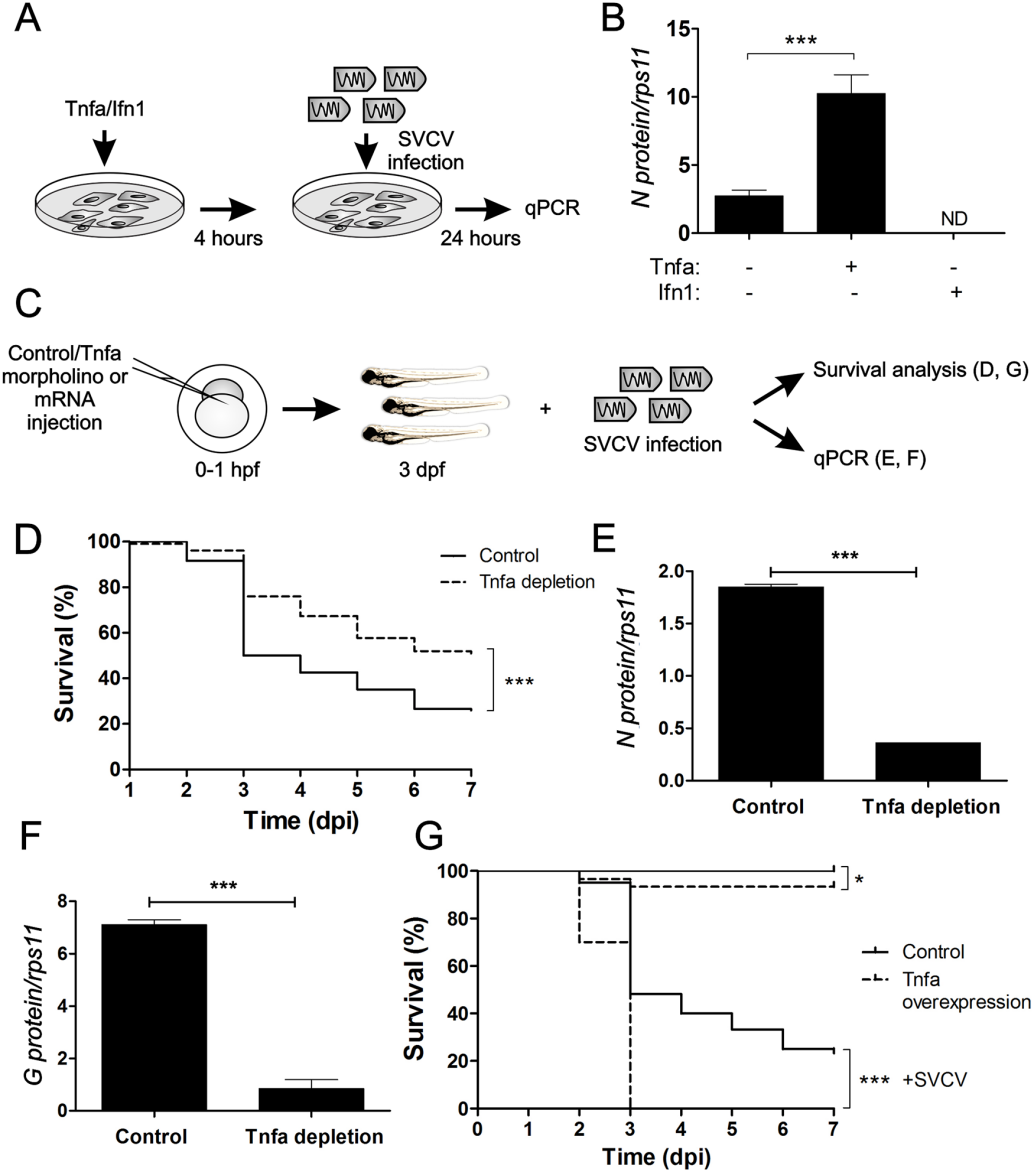
Tnfa enhances SVCV infection in zebrafish. (A) Workflow of the experimental design followed in (B). Recombinant zebrafish Tnfa or interferon 1 (Ifn1) were added to ZF4 cells growing in monolayer at 80% confluence and incubated for 4 hours. Subsequently, the medium was washed out and fresh medium containing SVCV was added. After 24 hours of incubation with the virus, the cells were harvested for qPCR analysis. (B) N protein mRNA expression levels assessed by qPCR relative to the housekeeping gene *rps11* and multiplied by 10^5^. Bars represent mean ± S.E.M. of indicated gene expression from one representative experiment. (C) Workflow of the experimental design followed in (D-G). Std (Control) or Tnfa mos (D-F) or antisense or Tnfa RNAs (G) were injected in zebrafish embryos at one-cell-stage of development. At 3 dpf, these larvae were immerse in RPMI containing inactivated SVCV (control) or intact SVCV for subsequently analysis of survival (D, G) or qPCR analysis at 48 hours post-infection (hpi) (E, F). Percentage of survival of Tnfa-depleted (D) and overexpressing (G) zebrafish larvae exposed to 10^9^ TCID_50_/ml SVCV. (E, F) The mRNA levels of the gene coding for the SVCV N protein as an estimation of the viral replication (E), and the RNA- levels of G protein (F) were determined in the infected larvae by qPCR in 10 pooled larvae at 48 hpi (5 dpf). The gene expression was normalized against *rps11* and multiplied by 10^5^ for N protein. Bars represent mean ± S.E.M. of triplicate readings from pooled larvae and the data are representative of two independent experiments. ***p<0.001. ND, not detected.

As in most infections, Tnfa is up-regulated in response to SVCV infection [12]. Unexpectedly, this up-regulation rather than help to control the infection, has a deleterious role in adult zebrafish [9]. To further study this phenomena, we first investigated whether or not Tnfa was also able to enhance SVCV replication both *in vivo* and *in vitro*. For that, we pre-incubated the zebrafish embryonic fibroblast cell line, ZF4, which expresses both Tnfrs [9], with zebrafish recombinant Tnfa or interferon 1 (Ifn1, also known as Ifnphi1) for 4 hours and, subsequently, the treated cells were infected with SVCV. At 24 hours post-infection (hpi), viral replication, measured as the presence of transcript of the nucleoprotein that forms the SVCV capside (N protein), was evaluated by RT-qPCR (Fig 1A). N protein transcripts significantly increased in Tnfa-treated cells and significantly decreased in Ifn1-treated cells (Fig 1B), suggesting that Tnfa enhances and Ifn1 decreases viral replication *in vitro*.

Both insufficient and excess Tnfa have been shown to promote susceptibility to mycobacterial infection [13]. We then asked whether endogenous rather than exogenous Tnfa was beneficial or detrimental to the host during SVCV infection. The percentage of animals that survived at 7 days post-infection (dpi) was significantly higher in Tnfa-depleted larvae when compared to controls (Tnfa expressing larvae) (55% versus 30%, respectively) (Fig 1C and 1D and S1A and S1B Fig). The survival percentage of control and Tnfa-depleted uninfected larvae was 100% in both cases. In accordance to these results, qPCR analysis of embryos harvested at 48hpi showed that the highest levels of viral replication (measured as the amount of SVCV N protein mRNA in infected animal tissues) (Fig 1E), and virus particles (measured as the amount of negative sense RNA encoding SVCV G glycoprotein in infected animal tissues) (Fig 1F), were found in control larvae. These results were further confirmed in larva forced to express Tnfa RNA, which showed drastic increased susceptibility to SVCV (Fig 1G and S1C and S1E Fig). Together, these results indicate that Tnfa enhances SVCV replication and pathogenesis *in vivo*.

### The Tnfa/Tnfr2 axis mediates increased SVCV replication in zebrafish

**Fig 2:**
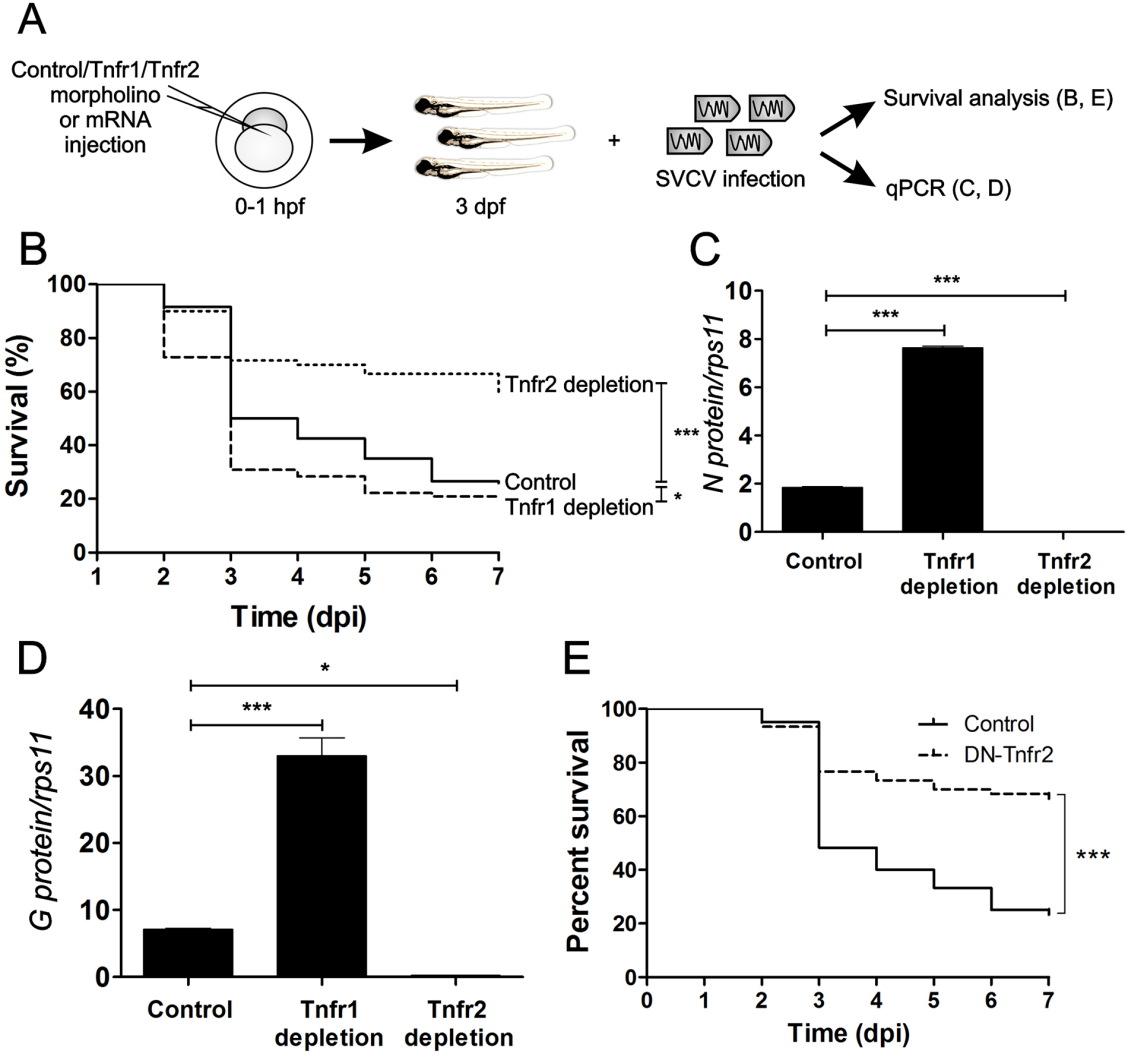
Tnfr2 mediates the Tnfa-triggered susceptibility of zebrafish to SVCV. (A) Workflow of the experimental design followed in (B-E). Std (Control), Tnfr1 or Tnfr2 mos (B-D) or antisense or DN-Tnfr2 RNAs (E) were injected in zebrafish embryos at one-cell-stage of development. At 3 dpf, these larvae were immerse in RPMI containing inactivated SVCV (control) or intact SVCV for subsequently analysis of survival (B, E) or qPCR analysis at 48 hours post-infection (hpi) (C, D). Percentage of survival of Tnfr-depleted (B) and DN-Tnfr2 overexpressing (E) zebrafish larvae exposed to 10^9^ TCID_50_/ml SVCV. (C,D) The mRNA levels of the gene coding for the SVCV N protein as an estimation of the viral replication (C), and the the RNA- levels of G protein (D) were determined in the infected larvae by qPCR in 10 pooled larvae at 48 hpi (5 dpf). The gene expression was normalized against *rps11* and multiplied by 10^5^ for N protein. Bars represents mean ± S.E.M. of triplicate readings from pooled larvae and the data are representative of two independent experiments. *p<0.1; ***p<0.001.

TNFα exerts its activity through the binding and activation of two receptors, TNFR1 and TNFR2 (Tumor necrosis factor receptor 1 and 2, respectively) [14]. Tnf receptors are expressed early during zebrafish development [15], and they both have important roles for the clearance of viral infections [16]. To further dissect the contribution of Tnfa signaling in SVCV pathogenesis, we performed loss-of-function experiments for both Tnfa receptors using specific antisense morpholinos (MOs) [15] in SVCV-infected embryos (Fig 2A and S1F–S1I Fig). Tnfr2-depleted larvae were distinctly more resistant to SVCV infection compared to their control siblings (60% versus 30%, respectively) (Fig 2B), while Tnfr1-depleted larvae showed a slightly, but statistically significant, reduced survival compare to their control siblings (Fig 2B). This result was supported by increased, or decreased, SVCV replication in Tnfr1- and Tnfr2-depleted larvae, respectively (Fig 2C). Accordingly, the presence of viral genomes was also higher in Tnfr1-depleted larvae and lower in Tnfr2-depleted larvae at 48 hpi (Fig 2D). In addition, larva forced to express a RNA encoding a dominant negative (DN) form of Tnfr2, which is lacking the entire intracellular signaling domain and extinguishes Tnfr2 signaling by trimerization with endogenous Tnfr2 [15], showed increased resistance to SVCV (Fig 2E and S1D and S1E Fig). Overall, these results suggest that Tnfa facilitates SVCV replication through Tnfr2 signaling.

#### Tnfa does not affect viral adhesion or viral delivery from endosomes to the cytosol

**Fig 3:**
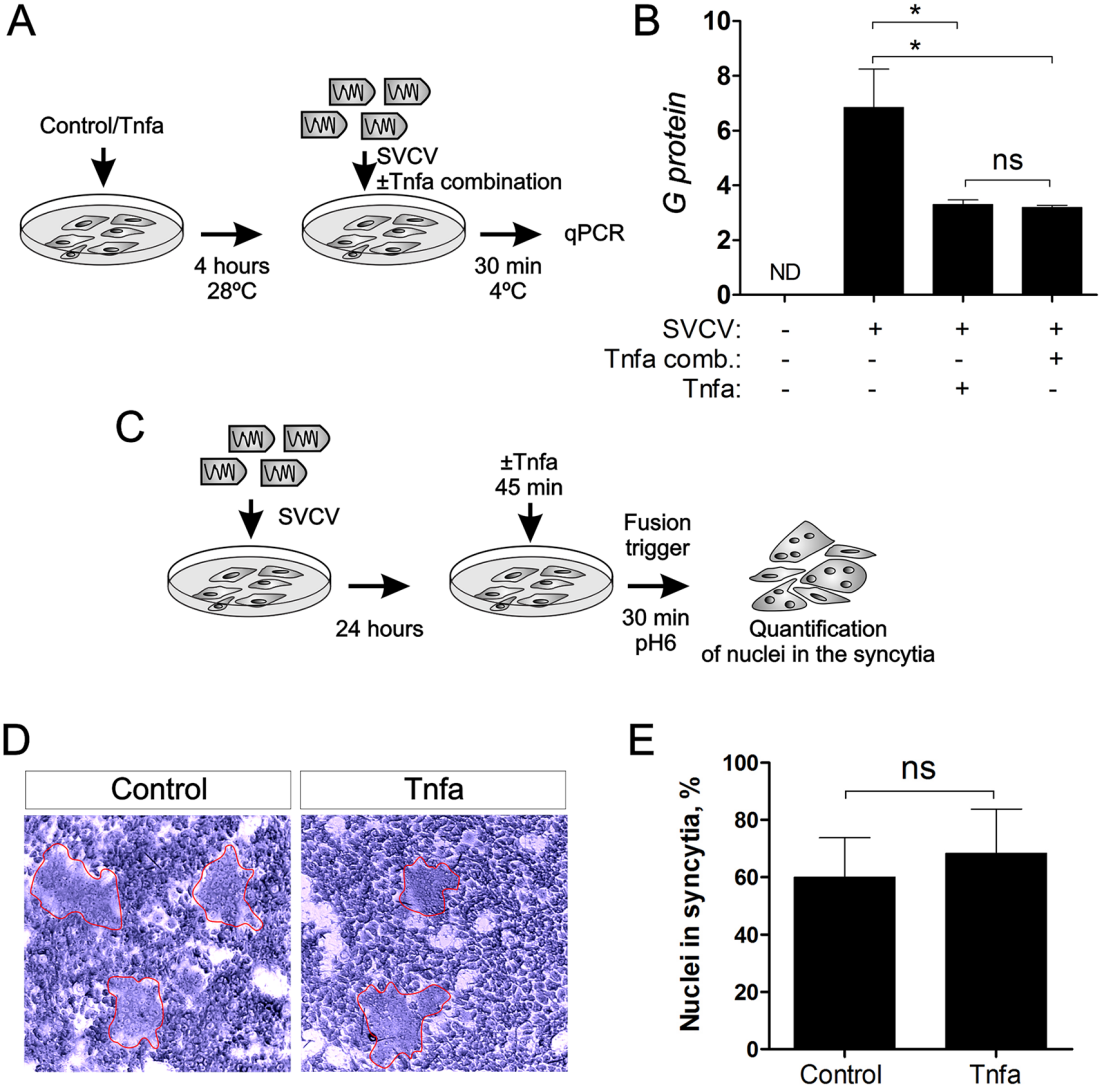
SVCV adhesion or syncytia production is not affected by Tnfa. (A) Workflow of the experimental design followed in (B). Briefly, recombinant zebrafish Tnfa was added to ZF4 cells growing in monolayer at 80% of confluence and incubated for 4 hours. Subsequently, the medium was washed out and new medium containing SVCV ± Tnfa (Tnfa combination) was added for 30 minutes at 4°C to allow virus adhesion but not virus internalization and replication. After 30 minutes, the cells were washed and harvested for qPCR analysis of the G protein gene for quantitation of the adhered virus. (B) qPCR analysis of the G protein encoding gene. Bars represent mean ± S.E.M. of triplicate readings from one sample and the data are representative of two independent experiments. *p<0.1. ns, non significant. Tnfa comb., Tnfa added in combination to the SVCV. (C) Workflow representing the experimental design for (D) and (E). ZF4 were incubated with SVCV for 24 hours. The medium containing virus was washed out and new medium containing Tnfa was added for 45 minutes. The fusion process was triggered by decreasing the pH to 6 for 30 minutes, and the nuclei in the syncytia were quantitated (D, E). Red lines denote syncytia.

To better understand the mechanism by which Tnfa enhances viral replication, we investigated the role of Tnfa in two key steps of SVCV pathogenesis, virus cell binding and the subsequent membrane fusion that allows virus release from the endosome to the cytosol. To interrogate if Tnfa could be facilitating the virus binding to the cell, we infected ZF4 cells for 30 minutes at 4°C to allow the virus adhesion but not its endocytosis and replication. Tnfa was added to ZF4 cells before or simultaneously to SVCV (Fig 3A). The number of viral particles adhered to Tnfa-treated cells (in both conditions, pre- or simultaneously added) was slightly lower than in the non-treated cells, assessed by qPCR of negative sense RNA encoding SVCV G glycoprotein (Fig 3B). This result suggests that Tnfa does not facilitate the SVCV cell binding.

After binding to the cell membrane, SVCV enters the cell by receptor-mediated endocytosis. Subsequently, these early endosomes are acidified after fusing to lysosomes. Endosomal acidification triggers conformational changes in the G protein of rhabdovirus, releasing the virus genome into the cytoplasm and allowing their replication [10,17,18]. In order to investigate whether or not this critical step in virus replication was affected by Tnfa, we performed a fusion assay [19] in SVCV-infected ZF4 cells pre-treated with Tnfa, where G-dependent cell fusion is triggered at pH = 6 (Fig 3C). The results show that the fusion process, evaluated by the number of nuclei in syncytia, in Tnfa-treated cells was unaffected compared to non-treated cells (Fig 3D and 3E). Taken together, these data indicate that Tnfa does not facilitate virus binding to the cell membrane or viral genome release into the cytoplasm.

### Tnfa does not antagonize the antiviral role of interferon during SVCV infection

**Fig 4:**
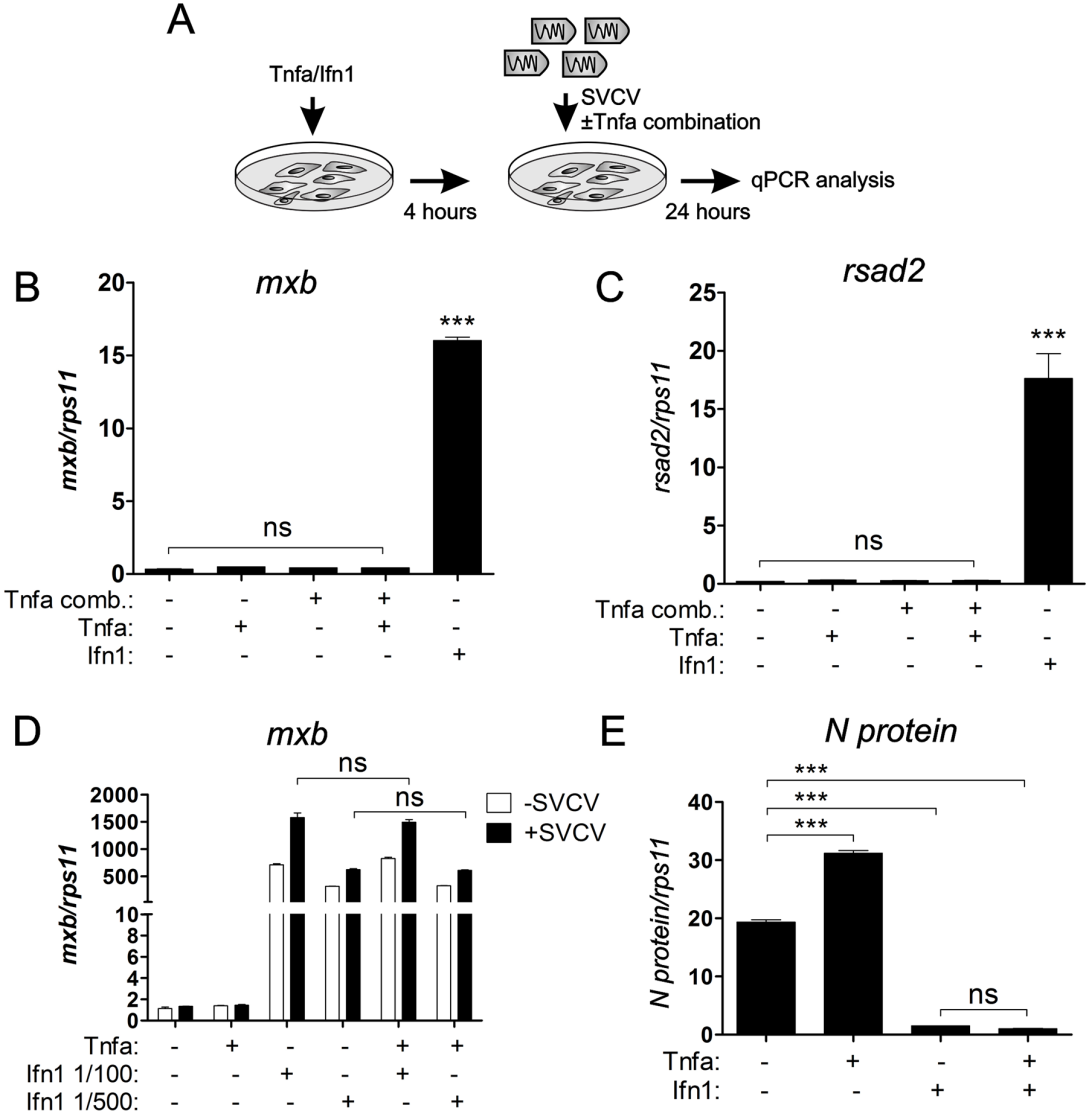
The antiviral role of interferon is not disrupted by Tnfa during SVCV infection. (A) Workflow representing the experimental design followed in (B-E). Briefly, Tnfa or Ifn1 were added to 80% confluent ZF4 cells and incubated for 4 hours. Subsequently, the medium was removed and fresh medium containing SVCV ± Tnfa was added during 24 hours for the following qPCR analysis from mRNA extracted from the cell. (B, C) mRNA levels of the genes encoding for the antiviral genes *mxb* (B) anf *rsad2* (C) of SVCV-infected ZF4 determined by qPCR. Gene expression is normalized against *rps11* and multiplied by 10 for *mxb* and 10^2^ for *rsad2*. Bars represent mean ± S.E.M. of triplicate readings from one sample and the data are representative of two independent experiments. (D) qPCR analysis of *mxb* expression levels in non infected or SVCV-infected cells previously treated with Tnfa or two different dilutions of Ifn1 (1/100 or 1/500). The gene expression is normalized against *rps11* and multiplied by 10^3^. Bars represent mean ± S.E.M. of triplicate readings from one sample and the data are representative of two independent experiments. (E) qPCR analysis of the N protein expression levels in SVCV-infected ZF4 cells previously treated with Tnfa or Ifn1. The gene expression is normalized against *rps11* and multiplied by 10^5^. Bars represent mean ± S.E.M. of triplicate readings from one sample and the data are representative of two independent experiments. ***p<0.001. ns, non significant.

Interferon is one of the most powerful antiviral cytokine [20]. It has been shown that interferons can act synergistically with TNFα to suppress virus replication [21,22]. However, TNFα and interferon can have antagonistic roles in certain cells such as human fibroblast-like synoviocytes [23]. Therefore, we decided to investigate if the enhancing role of TNFα in SVCV replication was the result of impairing the interferon response during SVCV infection. ZF4 cells were pre-treated with Tnfa and/or Ifn1 for 4 hours. Subsequently, these cells were infected with SVCV alone or in combination with Tnfa. After 24 hours, qPCR analysis was performed to detect the expression of antiviral host genes and viral N protein transcript (Fig 4A). The addition of Tnfa before or in combination with SVCV did not alter the transcript levels of the genes encoding major antiviral effectors, such as myxovirus (influenza) resistance b (Mxb), radical S-adenosyl methionine domain containing 2 (Rsad2), Mxc and protein kinase containing Z-DNA binding domains (Pkz) compared to untreated cells (Fig 4B and 4C and S2A and S2B Fig). In contrast, Ifn1-treated cells showed drastically increased levels of the transcripts for the same host genes (Fig 4B and 4C and S2A and S2B Fig). Cells were then pre-incubated with 2 different dilutions of Ifn1 (1/100 and 1/500), alone or in combination with Tnfa, and subsequent SVCV infection was performed. Both Ifn1 dilutions were able to increase the RNA levels of *mxb*, though these levels were unaffected by the simultaneous addition of Tnfa, in both uninfected and infected cells (Fig 4D). Altogether, these results indicate that Tnfa does not antagonize the antiviral role of Ifn1 during SVCV infection. To verify that Ifn1 was indeed interfering with SVCV replication in ZF4 cells, SVCV replication was quantitated by RT-qPCR analysis of the N protein transcripts in SVCV-infected ZF4 cells pre-treated with Tnfa and Ifn1 (Fig 4A and 4E). While the N protein mRNA levels were up-regulated in Tnfa-treated cells, they were down-regulated in the Ifn1, as well as in the Tnfa/Ifn1 combination (Fig 4E). This finding suggests that Ifn1 has a protective role against SVCV replication and that TNFα does not antagonize the antiviral role of Ifn1 during SVCV infection.

### Tnfa inhibits autophagy

**Fig 5:**
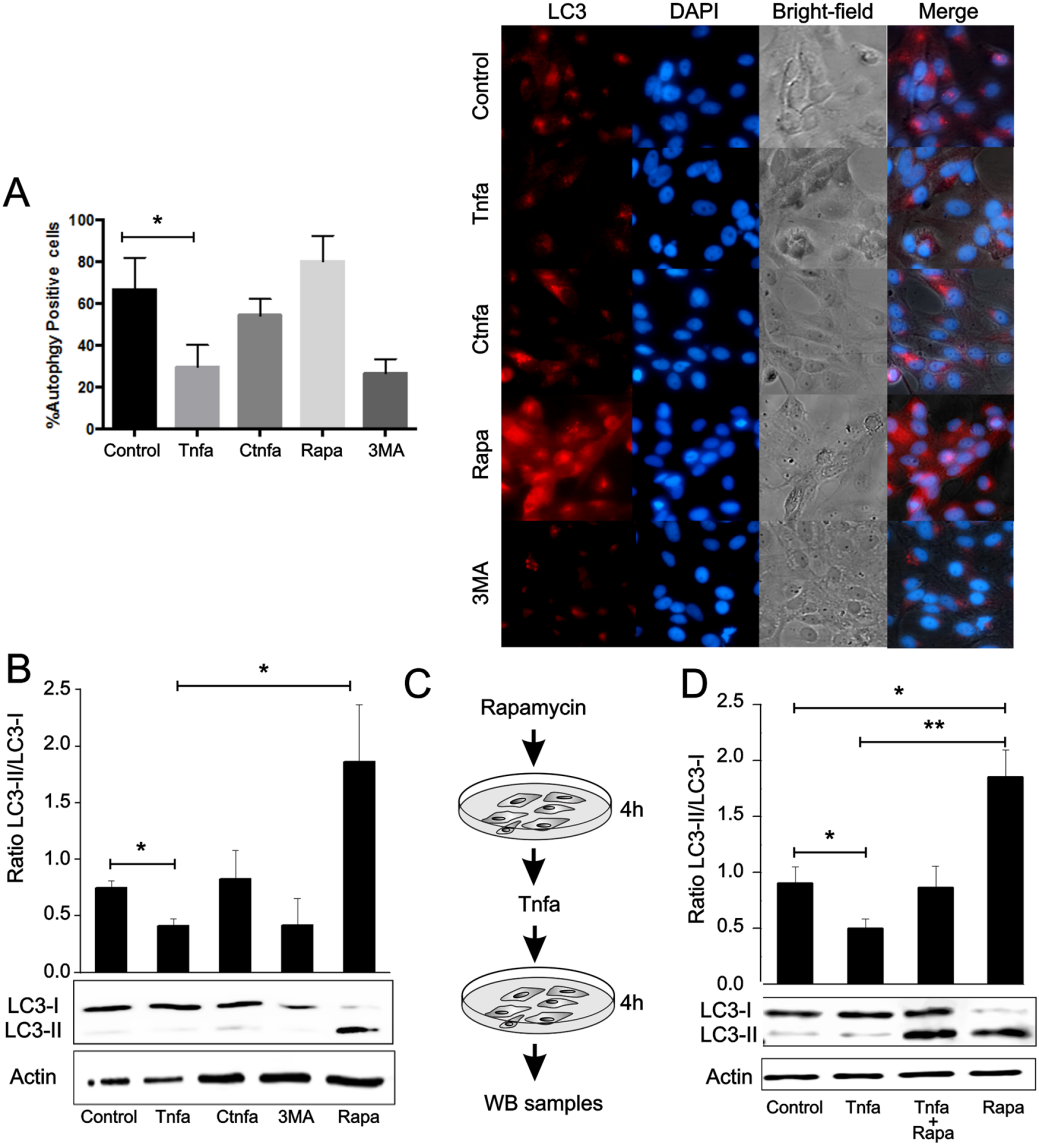
Tnfa inhibits autophagy. (A) ZF4 cells were treated with 0.1 μg of Tnfa or inactivated Tnfa (CTnfa), 1μM of Rapa, 10 mM of 3-methyladenine (3MA) or remain untreated. After 4 hours cells were fixed and then incubated with an antibody anti-LC3. Cells were finally stained with a fluorophore-conjugated secondary antibody (red fluorescence, LC3) and DAPI (blue, cell nuclei). The number of cells with LC3 puncta (n = 10) was determined (left panel). Bars represent mean ± S.E.M. Images are representative of the results obtained in 3 independent experiments (right panel). (B-D) Whole cell lysates were obtained from cells treated with Tnfa, CTnfa, Rapa or 3MA (B) or pre-treated with Rapa for 4 hours and then Tnfa or CTnfa was added for another 4 hours (C, D). LC3-I and LC3-II bands were visualized by WB using an anti-LC3 antibody and the protein content of the stained bands estimated by densitometry. The densitometry values were used to calculate LC3-II/ LC3-I ratios. Actin bands were detected as a protein load internal control using an anti-actin antibody. Data are shown as the mean±S.E.M. of 3 independent experiments. *p<0.05. **p<0.01.

Since autophagy is an efficient antiviral mechanism in response to many viral infections including SVCV [24,25], we asked if Tnfa could interfere with the autophagy-mediated clearance of SVCV by host infected cells. ZF4 cells were incubated with Tnfa for 4 hours and autophagy levels were assessed by cellular LC3 distribution. Cells were treated with autophagy modulators, such as 3-Methyladenine (3MA) and rapamycin (Rapa) to respectively inhibit or enhance autophagy. As expected, autophagy (red puncta indicating L3C recruitment) was clearly diminished in 3MA-treated cells and, in contrast, strongly increased in Rapa-treated cells (both in number and size of the autophagosomes) (Fig 5A). Interestingly, Tnfa treatment diminished autophagosome formation suggesting that Tnfa inhibits autophagy (Fig 5A). In contrast, cells treated with heat-inactivated Tnfa (control Tnfa, CTnfa) did not affect autophagy (Fig 5A).

After autophagy induction, the cytosolic soluble form of LC3 (LC3-I) is conjugated to phosphatidylethanolamine to form LC3-phosphatidylethanolamine conjugate (LC3-II), which is recruited to autophagosomal membranes. This process is conserved among vertebrates and present in mammals [26] and in fish [25]. Therefore, LC3-II/LC3-I ratio is commonly used to quantify autophagosome formation by western-blot (WB) [27]. In order to quantify autophagy formation in Tnfa-treated cells, western-blot for LC3 was performed from lysates of Tnfa-treated ZF4 cells at 4 hours post-treatment (Fig 5B). The LC3-II/LC3-I ratio decreased by 2-fold in Tnfa-treated cells, but was unaltered in heat-inactivated Tnfa (CTnfa), indicating the negative impact of Tnfa in autophagy (Fig 5B). As expected, the LC3-II/LC3-I ratio decreased after 3MA treatment and increased after Rapa addition (Fig 5B). To further verify the Tnfa-mediated down-regulation of autophagy, ZF4 cells were pre-treated with Rapa for 4 hours and, subsequently, Tnfa was added for 4 hours and western-blot for LC3 was performed using the cell lysates (Fig 5C). The addition of Tnfa to Rapa-treated cells led to a 2-fold reduction in the autophagy activity compared to cells treated with Rapa alone (Fig 5D). As expected, incubation of Tnfa alone reduced the autophagy activity by 2-fold compared to untreated cells (Fig 5D). All together, these results indicate that Tnfa reduces autophagy in ZF4 cells. Moreover, these data suggest a role for Tnfa as a potent effector in reverting autophagy after this process has been initiated.

### Tnfa impairs autophagy-mediated clearance of SVCV in ZF4 cells

**Fig 6:**
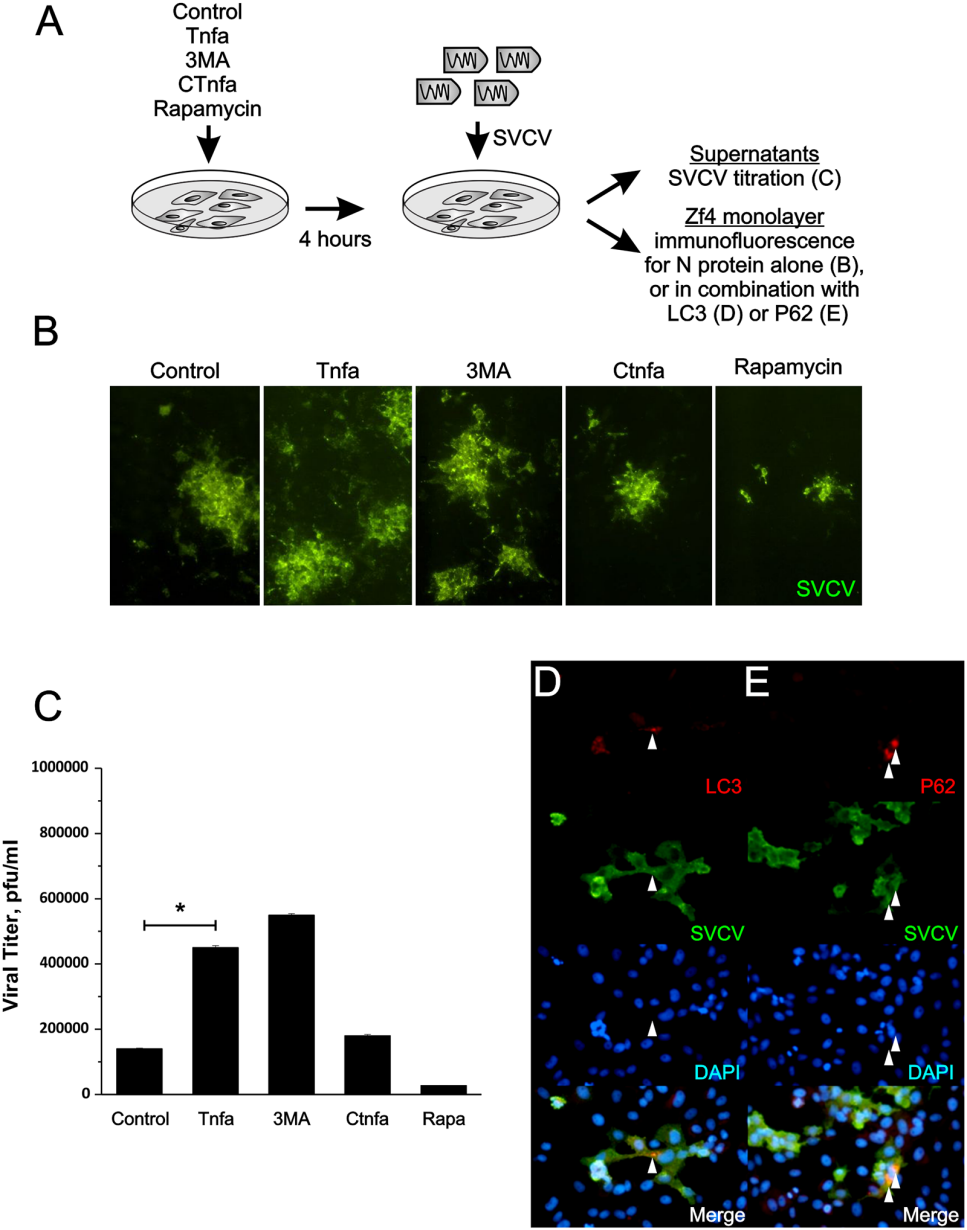
Tnfa increases the number of SVCV infective particles. (A) Workflow representing the experimental design followed in (B-E). Briefly, ZF4 monolayers were treated 0,1μg Tnfa or CTnfa 1μM of Rapa, 10 mM of 3-methyladenine (3MA) or remain untreated (control) for 4 hours and subsequently infected with a m.o.i. of 10^−2^ for 4 hours. (B-E) After 24 hours of infection, cells were fixed and stained with anti-SVCV antibody followed by the incubation of a FITC-labeled secondary antibody alone or combined with anti-LC3 (D) or anti-P62 (E) antibodies followed by a CF^™^594-labeled secondary antibody. (C) Virus titration in ZF4 cells in Plaque Forming Units per ml (PFU/ml) recovered from cell culture media of ZF4 pre-treated with Tnfa, CTnfa, 1μM of Rapa and 10 mM 3MA. Bars represent mean ± S.E.M. of three independent experiments. *p<0.05. (D,E) Colocalization of viral particles with autophagy markers (arrowheads) were observed and photographed with an inverted microscope.

We have previously demonstrated that autophagy has a protective role during SVCV and viral hemorrhagic septicemia virus (VHSV) infection [25]. To investigate whether Tnfa-mediated reduction of autophagy impairs SVCV clearance, ZF4 cells were pre-incubated with Tnfa prior to SVCV infection. As shown in the diagram of Fig 6A, the virus foci forming units (ffu) were first detected by immunofluorescence against N protein alone (Fig 6B), or in combination with LC3 (Fig 6D) or P62 (Fig 6E). To evaluate whether these ffu correlated with the infective viral particles, the SVCV present in the supernatant (viral yield) was also isolated and titrated by plaque forming units (PFU) (Fig 6A and 6C). The ffu number increased in Tnfa-treated cells compared to untreated and CTnfa-treated cells (Fig 6B). However, no differences on the foci size were found between these two treatments (Fig 6B). As expected, 3MA increased the ffu number, while Rapa decreased it (Fig 6B). Supernatant from cells treated with Tnfa contained 2.5-times more infective viral particles (4.5x10^5^ pfu/ml) than un-treated (1,8x10^5^ pfu/ml), or CTnfa-treated cells (1,4x10^5^ pfu/ml) (Fig 6C). As expected, 3MA-treatment also increased the SVCV pfu/ml (5,5x10^5^), while Rapa significantly decreased it (2,7x10^4^ pfu/ml) (Fig 6C). Notably, although viral particles colocalization with LC3 and P62 puncta was hardly observed in control cells, probably reflecting the rapid degradation/loss of immunogenicity of the virus, it was nicely observed in cells treated with Tnfa (Fig 6D and 6E). Taken together, these results demonstrate that Tnfa impairs viral clearance through the inhibition of the autophagy response in infected cells.

### Tnfa inhibits autophagy in vivo during SVCV infection in zebrafish larvae

**Fig 7:**
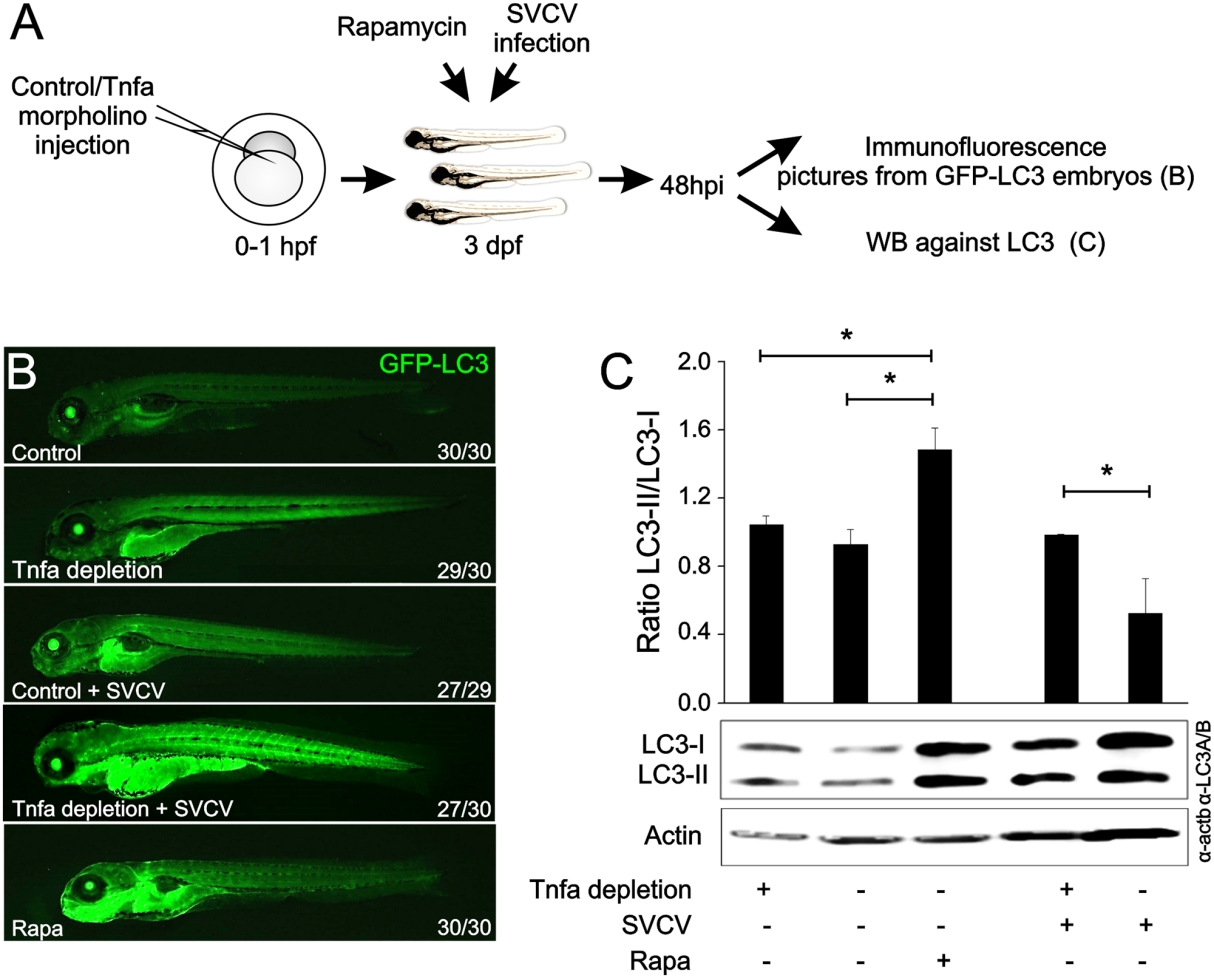
Tnfa inhibits the autophagy in zebrafish larvae. (A) Workflow representing the experimental design. B) Zebrafish GFP-LC3 transgenic embryos were injected with Tnfa or Std mos at the one-cell-stage of development. After 48 hours, a group of larvae injected with Std-mo was immersed in a bath with 1 μM Rapa and was freshly added every 24 h. The remaining larvae were divided in two and challenged by bath immersion with 10^9^ TCID_50_/ml SVCV SVCV or RPMI alone. After 72 hours of infection (5 dpf), larvae were collected, anesthetized with 0.16 mg/ml tricaine, mounted in 1% low melting point agarose supplemented with 0.16 mg/ml tricaine and images of the whole larvae taken using a Leica MZ16F fluorescence stereo microscope. Numbers in pictures represent the animals with the shown phenotype per total analyzed animals. (C) Zebrafish larvae were injected with morpholino (mo) Tnfa (Tnfa-MO) or Std (Std-mo) 1 hour post fertilization (hpf). After 36 hours, a group of larvae injected with Std-mo was immersed in a bath with Rapamycin and the remaining larvae (72 each group) were then divided in two and challenged by bath immersion with SVCV as above. After 48 hours of infection samples were recollected and LC3-I and LC3-II were detected by western-blot using an anti-LC3 antibody and the densitometry values were used to calculate LC3-II/ LC3-I ratios, represented as black bar graphs. Actin bands were detected as a protein load internal control using an anti-actin antibody. Data are shown as the mean±S.E.M. of 3 independent experiments. p<0.05.

To analyze the impact of Tnfa on the regulation of the host autophagic response to SVCV infection, we used a GFP-LC3 transgenic line that allows a real-time visualization of autophagy activity [28]. Morpholino-dependent Tnfa depletion resulted in increased basal autophagy in whole larvae, observed at low magnification as an increased fluorescence due to LC3 aggregation (Fig 7A and 7B). As expected, Rapa treatment also increased autophagy (Fig 7B). Moreover, as predicted from the previous *in vitro* data, SVCV-induced autophagy [25] was highly potentiated by depleting endogenous Tnfa [12](Lopez-Munoz et al., 2010)(Lopez-Munoz et al., 2010)(Lopez-Munoz et al., 2010)(Fig 7A and 7B). These results were confirmed by western blot analysis of the LC3-II/LC3-I ratio where depletion of Tnfa in infected larvae increased autophagy (Fig 7C). Therefore, Tnfa inhibits autophagosome formation during viral infection *in vivo*.

## Discussion

Although the administration of anti-TNFα therapies normally aggravates viral infections, there are a few reports suggesting that TNFα inhibition could be beneficial for the treatment of certain viral infections [5]. However, the mechanism by which viruses manipulate the host-produced TNFα for their own benefit had never been determined. Here, we have used the zebrafish as an infection model to examine *in vitro* and *in vivo* the mechanisms by which TNFα enhances viral pathogenesis. We utilized the previously established viral infection model of SVCV in zebrafish, in which excess Tnfa had already been reported to increase viral susceptibility [9], to dissect the possible negative role of TNFα for the host during SVCV infection. Our studies demonstrate that Tnfa enhances SVCV replication through its receptor Tnfr2. Mechanistically, Tnfa does not alter SVCV binding to the cells, its escape from the endosome to the cytosol, or the Ifn-mediated antiviral response. In contrast, Tnfa inhibits autophagy both *in vitro* and *in vivo*, leading to decreased viral clearance and, consequently, to a higher susceptibility to the infection.

The increased survival of Tnfa- and Tnfr2-depleted larvae infected with SVCV demonstrates that Tnfa signaling through Tnfr2 has a deleterious effect in the host during SVCV infection. These results are further confirmed by the increased susceptibility of larvae forced to express Tnfa, confirming previous studies using recombinant Tnfa [9], and by the increased resistance of larvae forced to express a DN form of Tnfr2. The fact that the percentage of survival of Tnfr1-depleted larvae is slightly reduced compared to control larvae suggests that Tnfa signaling through Tnfr1 might have some protective role against SVCV infection. Furthermore, the observation that Tnfr2 depletion leads to a much higher larval survival than Tnfa depletion (70% versus 55%, respectively), further supports dual roles for Tnfa during viral infection, being protective signaling through Tnfr1 and detrimental signaling through Tnfr2. However, the overall effect of Tnfa during viral infection is predominately harmful for the host. Therefore, we need to be aware that the manipulation of each Tnf receptor leads to different outputs than Tnfa depletion alone. Thus, the use of specific TNF receptor inhibitors, rather than TNFα neutralizing drugs, could prove to be beneficial for the treatment of TNFα-related pathologies [15,29]. The potential protective role of signaling through Tnfr1 during SVCV infection still remains unexplored, and further experiments should be performed to investigate this phenomenon.

Since this is the first study conducted to address the enhancing role of TNFα in viral pathogenesis, we decided to dissect the essential steps occurring during viral infection in order to identify which of them, if any, were affected by TNFα. These steps include virus adherence to the cell, release from the endosome to the cytosol, replication and new viral particle formation. Our studies demonstrate that Tnfa slightly reduces SVCV binding to the ZF4 cells yet the fact that this modest reduction is also observed when Tnfa is added simultaneously with the SVCV, suggests that Tnfa could be physically interfering with the SVCV rather than deterring its adhesion through the TNFα activation pathway. In addition, we also demonstrate that Tnfa does not affect the SVCV capability to escape from the endosome to the cytosol.

Here, we have characterized the possible interference of TNFα in two key antiviral cell mechanisms that restrict virus replication: interferon response [30] and autophagy [31]. Our studies demonstrate that while Tnfa does not alter the interferon response during SVCV infection, it is able to diminish the viral-induced autophagic cell response *in vitro* and *in vivo*. Although TNFα has generally been linked to an up-regulation of autophagy [32–35], it has also been shown that, in certain contexts, TNFα up-regulates mTOR activity through NF-κB, leading to autophagy inhibition [36]. In agreement with this, we provide evidences that Tnfa inhibits autophagy, which leads to increased viral susceptibility. Interesting, TNFα can also have a dual role in viral infection by promoting cell survival or cell death depending on the expression and activation balance of its receptors [37]. Although further studies should be conducted to address whether the TNFα/TNFR2 axis indeed inhibits autophagy through the activation of NF-κB, this is quite plausible since Tnfr2 mainly regulates NF-κB activation in zebrafish larvae [15,29]. It would be of interest to inhibit TNFα, or potentially TNFR2, in SVCV-infected carps for the treatment of this viral disease that produces abundant losses in aquaculture worldwide. In addition, it would be advantageous to manipulate the activation of TNF receptors in those viral infections in which autophagy plays an antiviral role, such as HSV1, HIV-1, Sindbis virus, chikungunya virus and West Nile virus [38]. It is important to emphasize that anti-TNFα therapies have already been suggested to be helpful for the treatment of some of these aforementioned viral infections, such as HIV-1 [5]. This therapeutic approach could have important health implications for the treatment of these devastating viral infections since, to date, there are no available treatments for the majority of them.

## Materials and Methods

### Ethics statement

The experiments performed comply with the Guidelines of the European Union Council (86/609/EU) and the Spanish RD 53/2013. Experiments and procedures were performed as approved by the Bioethical Committee of the University of Murcia (approval number #537/2011).

### Cell lines and virus

The fish cell line ZF4 (zebrafish embryonic fibroblast) was purchased from the American Type Culture Collection (ATCC, #CRL-2050). Cells were maintained at 28°C in a 5% CO_2_ atmosphere in RPMI-1640 Dutch modified (Gibco) cell culture medium containing 10% fetal bovine serum (FBS) (Sigma, F6178), 1 mM pyruvate (Gibco), 2 mM L-glutamine (Gibco), 50 μg/mL gentamicin (Gibco) and 2 μg/mL fungizone (Gibco).

The SVCV isolate 56/70 (kindly provided by Dr. P. Fernández-Somalo, Laboratorio Central de Veterinaria, MAGRAMA) was propagated in ZF4 cells at 22°C as previously described [39]. Supernatants from SVCV-infected cell monolayers were clarified by centrifugation at 4,000 × g for 30 min and kept in aliquots at −80°C. Clarified supernatants were used for the experiments. The virus stock was titrated in 96-well plates by limit-dilution (50% tissue culture infectious dose (TCID_50_)/ml) [40].

### Zebrafish husbandry

The zebrafish (*Danio rerio* H.) AB strain was obtained from the Zebrafish International Resource Center (ZIRC, https://zebrafish.org/home/guide.php). The transgenic line *Tg(CMV*:*EGFP-map1lc3b)* ^*zf155*^ (GFP-LC3 for simplification) was previously described [28]. Fish were mated, staged, raised, and processed as previously described [41].

### Morpholino and RNA injection and pharmacological treatments

In vitro-transcribed RNA of wild type Tnfa and DN Tnfr2 [15] was obtained following manufacturer’s instructions (mMESSAGE mMACHINE kit, Ambion). Morpholinos were diluted in DEPC-treated water at a concentration of 0.3 mM (Standard-mo, Gene Tools) 0.5 mM (Tnfa-MO, 5’-GCAGGATTTTCACCTTATGGAGCGT-3’ [42], 0.65 mM (Tnfr1-mo, 5’-ctgcattgtgacttacttatcgcac-3’ [15], 0.3 mM (Tnfr2-mo, 5’-ggaatctgtgaacacaaagggacaa-3’ [15]. Morpholinos and RNA were mixed in microinjection buffer and microinjected into the yolk sac of one-cell-stage embryos using a microinjector (Narishige) (0.5–1 nl per embryo). The same amount of MOs and/or RNA were used in all experimental groups. The efficiency of the MOs was checked by RT-PCR [15,42].

### *In vivo* viral infection assays

Groups of 20–40 wild type or GFP-LC3 transgenic zebrafish larvae of 3 days post fertilization (dpf) were challenged at 26°C by bath immersion in 5 ml of filtered egg water (60 mg/ml sea salts in distilled water) containing ~10^9^ TCID_50_ (50% tissue culture infectious dose)/ml SVCV. Twenty four hours later, the solution containing the larvae was diluted by adding 35 ml of egg water and the larvae were monitored every 24 hours for 8 days for clinical signs of disease and mortality. Fifteen pooled larvae were collected at 48 hpi in 250 μl Trizol (15 larvae) for gene expression studies. For *in vivo* visualization of autophagy activity, GFP-LC3 transgenic larvae were anesthetized at 48 hpi (5 dpf) with 0.16 mg/ml tricaine and mounted in 1% low melting point agarose supplemented with 0.16 mg/ml tricaine. Images of the whole larvae were then taken using a Leica MZ16F fluorescence stereo microscope. As positive control, 48 hpf larvae were treated with 1 μM Rapa (Calbiochem) for 72 h.

### *In vitro* viral infection assays

The SVCV infectivity *in vitro* was evaluated by two different methods, RT-qPCR and foci forming unit assays. To detect SVCV by RT-qPCR, ZF4 cells were cultured in 25 cm2 flasks at 80% confluence and treated with 100 ng/ml of zebrafish recombinant Tnfa [9] or Ifn1 (dilutions 1/100 or 1/500) [43] for 4 hours at 28°C and 5% CO2. Subsequently, the media was removed, cells were washed twice with the cell media containing 2% FBS and infected with SVCV (multiplicity of infection (MOI) of 10^−3^) in the presence or in absence of Tnfa (100 ng/ml) at 22°C for 24 hours. Afterward, the media was removed, RNA extracted, cDNA obtained and qPCR carried out as below indicated. Two different sets of primers (S1 Table) were used for SVCV detection: i) to quantify virus replication a primer pair amplifying the mRNA of N protein of SVCV and ii) to quantify the amount of viral genomes (negative sense RNA), a primer pair designed to detect the negative sense RNA encoding the gen of SVCV G protein.

For foci forming unit assays a previously developed methodology [44] with minor modifications was used. Briefly, ZF4 cells, grown on 96-well plates, were treated with 0.1 μg/ml or 1 μg/ml Tnfa, 1 μg/ml heat inactivated (C Tnfa), 1 μM RAP or 10 mM 3MA at 28°C for 4 hours. After incubation, cell culture medium was removed and cells were infected with SVCV (multiplicity of infection (MOI) of 10–2) at 22°C. Two hours post-infection, the supernatants from infected cell cultures were removed to eliminate non-bound virus, cell media containing 2% FBS added and plates further incubated for 24h. On the one hand, supernatants form infected cells were harvested and stored at -80°C for viral tritation to determine the virus yield as below indicated. On the other hand, cell were fixed with a solution of 4% formaldehyde (Sigma, F1635) for 15 min, washed with PBS and further fixed with cold methanol (−20°C) for 15 min. Fixed cells were stained with a monoclonal antibody to SVCV (Teknokroma Analítica S.A. monoclonal antibody anti-SVCV) at 4°C for 24h [45]. After washing with PBS and cell monolayers were incubated with a FITC-labelled rabbit anti-mouse antibody (SIGMA) diluted 1/500 and incubation was continued for 30 min. Stained SVCV infected cell foci were then viewed and photographed with an inverted fluorescence microscope (Nikon Eclipse TE2000-U; Nikon Instruments, Inc., NY) provided with a digital camera (Nikon DS-1QM, Nikon Instruments, Inc., NY). At least, three different assays, each in duplicated, were performed

### Viral yields

Virus titers in the supernatants of SVCV infected cells in the presence or absence of Tnfa were determined by a plaque forming units assay [39] and expressed as plaque forming units (PFU) per ml. Briefly, different dilutions of each supernatant (from 10^−3^ to 10^−9^) were added to ZF4 cell monolayers, grown on 24-well plates at 22°C for 2 hours. Then, culture media was removed and the infected cell monolayers covered with a solution of RPMI-1640 cell culture medium with 2% FCS and a 2% aqueous solution of methyl cellulose (Sigma). Cell plates were incubated at 22°C for 5 days and then the media with methyl cellulose was removed. Finally, wells were stained with crystal violet-formalin and plaques counted.

### Viral binding assays

To analyze whether or not TNFα impairs the binding of SVCV viral particles to target cells, ZF4 cells grown in 25 cm^2^ flasks at 80% confluence, were treated with TNFα (100 ng/ml) for 4 hours at 28°C. The media was then removed, cells were washed twice with the cell media containing 2% FBS and infected with SVCV (10^−3^ MOI) in the presence or absence of TNFα (100 ng/ml) for 30 minutes at 4°C to allow virus binding/attachment but not its endocytosis. Afterward, the media was removed, cells washed twice with cell media containing 2% FBS, RNA extracted and cDNA obtained. By means of qPCR using specific primers (S1 Table) the presence of SVCV G protein in the surface of the infected cells (viral binging) was evaluated.

### Fusion assays

ZF4 cells, grown on 96 well-plates, infected with SVCV (MOI of 10^−2^). Two hours post-infection, the supernatants from infected cell cultures were removed to eliminated un-bound virus and fresh cell culture medium 2% FBS was added. After 24h of incubation at 22°C, the cell culture medium was removed and the SVCV-infected cell monolayer treated with Tnfa (100 ng/ml) for 45 min. The cells were then washed and the membrane fusion triggered by incubating the cells with fusion medium [44] at pH 6 for 30 min at 22°C. After that, cell monolayers were washed and subsequently incubated with fusion medium at pH 7.5 for 2 h at room temperature. Finally, cells were fixed with cold methanol (-20°C) for 15 min, dried and stained with Giemsa (5 mg/ml in PBS). Cells were viewed and photographed with an inverted fluorescence microscope (Nikon) provided with a digital camera (Nikon DS-1QM). At least, three different assays, each in duplicated, were performed.

### Western blot

ZF4 cells were grown on 24-well plates in culture medium supplemented with 10% FBS at 28°C. After 24 h, the different treatments (1 μM RAP, 10 mM 3MA, Tnfa (100 ng/ml) or CTnfa (100 ng/ml) were added. After 4 hours of incubation, culture media was removed and cell monolayers were resuspended in 500 μl of PBS with a cocktail of protease inhibitors (Sigma). Cells were then processed to a frozen/thawed cycle 4 times and protein concentration adjusted before loading protein samples onto the gel. Samples were then loaded in Tris—Glycine sodium dodecyl sulfate 17% polyacrylamide gels under reducing conditions and the electrophoresis performed at 100 V for 90 min. The proteins in the gel were then transferred to nitrocellulose membranes (BioRad) for 75 min at 100 V in transfer buffer (2.5 mM Tris, 9 mM glycine, 20% methanol). The membranes were then blocked with 8% dry milk, 0.05% Tween-20 in PBS. Then, the membranes were incubated with the primary antibody microtubule-associated protein 1 light chain-3 (LC3)-I/LC3-II, a polyclonal antibody anti-LC3A/B (Cell Signaling Technology) diluted 1000-fold in PBS containing 5% BSA and 0.1% Tween-20 as indicated by the manufacturer. Membranes were then washed 3 times with PBS containing 0.05% Tween-20 for 15 min before incubation with GAR-Po in 0.5% milk in PBS for 90 min. After the last 3 washes with PBS containing 0.05% Tween-20, the peroxidase activity was detected by using ECL Select chemiluminescence reagents (Amersham Biosciences, RPN2232) and revealed by exposure to X-ray. Protein bands were analyzed by densitometry using the Totalab Software. Analysis of LC3-I and LC3-II bands was performed and calculated as relative to the actin intensity band. Results are presented as the ratio of LC3-II/LC3-I from 3 independent experiments.

### Immunofluorescence assays

After 4 hours of incubation with the different treatments (1 μM RAP, 10 mM 3MA, Tnfa (100 ng/ml) or CTnfa), monoloayers were fixed with a solution of 4% formaldehyde (Sigma) for 15 min, washed with PBS and further fixed with cold methanol (−20°C) for 15 min. Cell monolayers were then incubated overnight at 4°C with the anti-LC3 or anti-p62 (Abcam) antibodies in dilution buffer (PBS with 0.03% Triton X 100 [Sigma]) and 5% of albumin from bovine serum (BSA, Sigma). To visualize LC3 and p62, monolayers were washed again and incubated with appropriate secondary antibodies (in dilution buffer) for 1 h. To visualize nuclei, cells were stained with 1 μg/mL of 4′-6-Diamidino-2-phenylindole (DAPI) for 10 min. Cell monolayers were finally washed for another 3 times. Cells were viewed and photographed with an inverted fluorescence microscope (Nikon Eclipse TE2000-U; Nikon Instruments, Inc., NY) provided with a digital camera (DS-1QM, Nikon Instruments, Inc., NY).

### RNA isolation, cDNA synthesis and RT-qPCR assays

Total mRNA was extracted from pooled larvae or ZF4 cells with TRIzol Reagent (Life Technologies) and purified using the PureLink RNA Mini Kit (Life Technologies) following the manufacturer’s instructions. Isolated RNAs were stored at −80°C until used. The purified mRNA was treated with DNase I, amplification grade (1 unit/μg RNA; Invitrogen). SuperScript III RNase H− ReverseTranscriptase (Invitrogen) was used to synthesize the first strand of cDNA with an oligo-dT18 primer from 1 μg of total RNA at 50°C for 50 minutes. Real-time PCR was performed with an ABI PRISM 7500 instrument (Applied Biosystems) using SYBR Green PCR Core Reagents (Applied Biosystems). Reaction mixtures were incubated for 10 minutes at 95°C, followed by 40 cycles of 15 seconds at 95°C and 1 minute at 60°C, and finally by 15 seconds at 95°C, 1 minute 60°C and 15 seconds at 95°C. For each mRNA, gene expression was normalized to the ribosomal protein S11 (*rps11*) content in each sample using the Pfaffl method [46]. In all cases, the PCR was performed with triplicate samples and repeated with at least two independent samples. The primers used are shown in S1 Table.

### Statistical analysis

Data are shown as mean ± SEM of at least three separate assays for gene expression experiments. Data were analyzed by ANOVA and a Tukey multiple range test to determine differences between groups, while the differences between two samples were analyzed by the Student t test. Log-rank (Mantel-Cox) Test was used for the survival curves.

## Supporting Information

S1 Table Primers used in this study. The gene symbols followed the Zebrafish Nomenclature Guidelines http://zfin.org/zf_info/nomen.html). ENA, European Nucleotide Archive.
(DOCX)

S1 Fig Related to Figs 1 and 2. Validations of the loss- and gain-of-function experiments used in this study. (A, B and F-I) RT-PCR analysis of Tnfa (A, B) and Tnfr1 (F, G) and Tnfr2 mos (F, I) induced altered splicing of the *tnfa*, *tnfr1* and *tnfr2* transcripts, respectively at 3 dpf. The annealing of mos (solid lines), the primers used for the amplification (arrowheads) and the inframe premature stop codons (asterisks) are indicated. (A, B) A 740 bp product with an intact intron inserted between exons 1 and 2 of *tnfa* was only observed in samples injected with Tnfa MO, while the same was absent from standard mo-injected fish. (F, G) A 540 bp product containing a deletion of the last 16 bp of exon 6 of *tnfr1* transcript was observed in samples injected with Tnfr1 MO, while it was absent from standard mo-injected fish. This deletion resulted in a predicted Tnfr1 protein lacking the signaling domain. (H, I) A 611 bp product containing a deletion of whole exon 2 of *tnfr2* transcript was observed in samples injected with Tnfr2 mo, while it was absent from standar mo-injected fish. This deletion resulted in a predicted Tnfr2 protein lacking most extracellular domain and the whole signaling domain. (C-E) RT-qPCR analysis of 2 dpf larvae forced to express Tnfa (C) and DN-Tnfr2 (D), and amplicon obtained for the housekeeping gene *actb* (E).
(TIF)

S2 Fig Related to Fig 4: The antiviral role of interferon is not disrupted by Tnfa during SVCV infection. mRNA levels of genes encoding the antiviral genes *mxc* (A) and *pkz* (B) of SVCV-infected ZF4 cells pre-treated with Tnfa or Ifn1, or Tnfa treatment in combination (Tnfa comb.) to SVCV infection determined by qPCR. The gene expression is normalized against *rps11* and multiplied by 10^4^ for *mxc* and 10^2^ for *pkz*. Bars represent mean ± S.E.M. of triplicate readings from one sample and the data are representative of two independent experiments. ***p<0.001. ns, non significant.
(TIF)
