# Supplementary material for: TNFα Impairs Rhabdoviral Clearance by Inhibiting the Host Autophagic Antiviral Response
Source: PLoS Pathog. 2016 Jun 28;12(6):e1005699. doi: 10.1371/journal.ppat.1005699 (PMC4924823; doi:10.1371/journal.ppat.1005699)
Supplement: S1 Table — The gene symbols followed the Zebrafish Nomenclature Guidelines http://zfin.org/zf_info/nomen.html). ENA, European Nucleotide Archive. (DOCX) [file ppat.1005699.s001.docx]

| **Gene** | **ENA or ENSEMBL accession numbers** | **Nucleotide sequence (5’→3’)** | | **Use** |
| --- | --- | --- | --- | --- |
| **ZEBRAFISH PRIMERS** | | | | |
| *mxb* | NM_001128672 |  | AATGGTGATCCGCTATCTGC | RT-qPCR, gene expression |
|  |  |  | TCTGGCGGCTCAGTAAGTTT |  |
| *mxc* | NM_001007284 |  | GAGGCTTCACTTGGCAACTC |  |
|  |  |  | TTGTTCCAATAAGGCCAAGC |  |
| *pkz* | NM_001040376 |  | GGAGCACCGTACAGGACATT |  |
|  |  |  | CTCGGGCTTTATTTGCTCTG |  |
| *rsad2* | EF014961 |  | AGCAGATCACCGCTCTCAAT |  |
|  |  |  | CCAGACACTGGATGCTCTGA |  |
| *rps11* | NM_213377 |  | TAAGAAATGCCCCTTCACTG |  |
|  |  |  | GTCTCTTCTCAAAACGGTTG |  |
| *tnfa* | ENSDARG00000009511 |  | GCGCTTTTCTGAATCCTACG | RT-qPCR, *tnfa* validation |
|  |  |  | TGCCCAGTCTGTCTCCTTCT |  |
|  |  |  | TGGATGAAGCTTGAGAGTCGGGG | RT-PCR, MO validation |
|  |  |  | TGCCCAGTCTGTCTCCTTCT |  |
| *tnfr1* | ENSDARG00000018569 |  | AAAGGATCCTGGATGAGGATATGTCAACTGACC |  |
|  |  |  | TGCTGGTTTTGCATAGGTGA |  |
| *tnfr2* | ENSDARG00000070165 |  | AAAGGATCCTGGATGACGGTGGTGTGGCTCTTGGCG |  |
|  |  |  | AAAGGGCCCGGGTCAGGCGGAGGGTTGCTTCGGATC |  |
|  |  |  | GAAGGAAAGATTCCTCCGCCA | RT-qPCR, *DN-tnfr2* validation |
|  |  |  | GTCCCTGGTTTGCATCTACTGCA |  |
| **SVCV PRIMERS** | | | | |
| *N protein* | NC_002803 | TGAGGTGAGTGCTGAGGATG | | RT-qPCR,  viral load (RNA+) |
|  |  | CCATCAGCAAAGTCCGGTAT | |  |
| *G protein* | NC_002803 | TACAGATTCGGGGGATCTTG | | RT-qPCR,  viral load (RNA-) |
|  |  | ACCAACGTTCCATCAACACA | |  |
