# Supplementary figures and images for: TNFα Impairs Rhabdoviral Clearance by Inhibiting the Host Autophagic Antiviral Response
Source: PLoS Pathog. 2016 Jun 28;12(6):e1005699. doi: 10.1371/journal.ppat.1005699 (PMC4924823; doi:10.1371/journal.ppat.1005699)

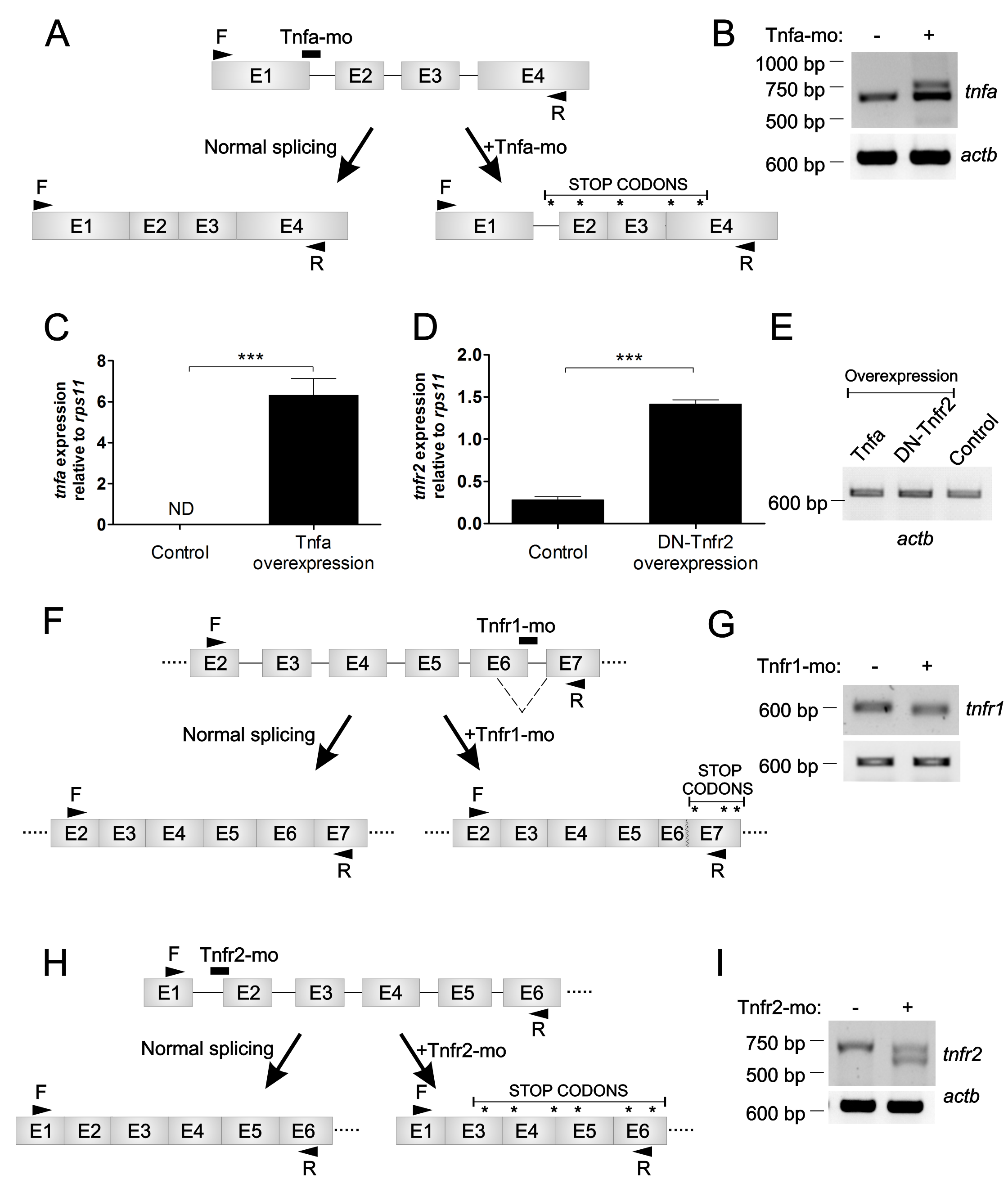

Supplement: S1 Fig — (A, B and F-I) RT-PCR analysis of Tnfa (A, B) and Tnfr1 (F, G) and Tnfr2 mos (F, I) induced altered splicing of the tnfa, tnfr1 and tnfr2 transcripts, respectively at 3 dpf. The annealing of mos (solid lines), the primers used for the amplification (arrowheads) and the inframe premature stop codons (asterisks) are indicated. (A, B) A 740 bp product with an intact intron inserted between exons 1 and 2 of tnfa was only observed in samples injected with Tnfa MO, while the same was absent from standard mo-injected fish. (F, G) A 540 bp product containing a deletion of the last 16 bp of exon 6 of tnfr1 transcript was observed in samples injected with Tnfr1 MO, while it was absent from standard mo-injected fish. This deletion resulted in a predicted Tnfr1 protein lacking the signaling domain. (H, I) A 611 bp product containing a deletion of whole exon 2 of tnfr2 transcript was observed in samples injected with Tnfr2 mo, while it was absent from standar mo-injected fish. This deletion resulted in a predicted Tnfr2 protein lacking most extracellular domain and the whole signaling domain. (C-E) RT-qPCR analysis of 2 dpf larvae forced to express Tnfa (C) and DN-Tnfr2 (D), and amplicon obtained for the housekeeping gene actb (E). (TIF) [file ppat.1005699.s002.tif]

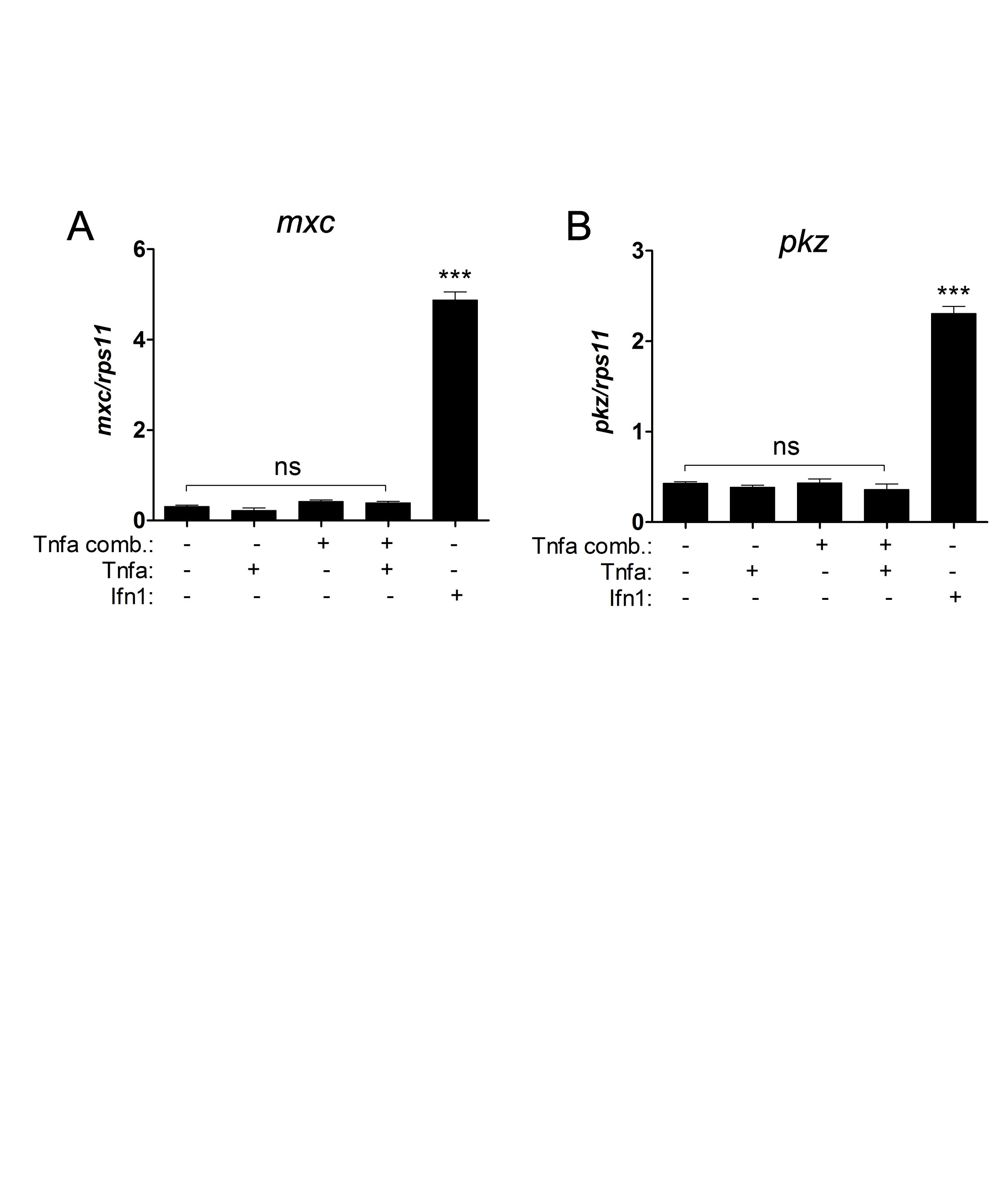

Supplement: S2 Fig — mRNA levels of genes encoding the antiviral genes mxc (A) and pkz (B) of SVCV-infected ZF4 cells pre-treated with Tnfa or Ifn1, or Tnfa treatment in combination (Tnfa comb.) to SVCV infection determined by qPCR. The gene expression is normalized against rps11 and multiplied by 104 for mxc and 102 for pkz. Bars represent mean ± S.E.M. of triplicate readings from one sample and the data are representative of two independent experiments. ***p<0.001. ns, non significant. (TIF) [file ppat.1005699.s003.tif]
